# Existence of periodic solution for fourth-order generalized neutral *p*-Laplacian differential equation with attractive and repulsive singularities

**DOI:** 10.1186/s13660-018-1849-x

**Published:** 2018-09-24

**Authors:** Yun Xin, Hongmin Liu

**Affiliations:** College of Computer Science and Technology, Henan Polytechnic University, Jiaozuo, China

**Keywords:** 34C25, 34K13, 34K40, Positive periodic solution, *p*-Laplacian, Fourth-order, Attractive and repulsive singular, Generalized neutral operator

## Abstract

In this paper, we investigate the existence of a positive periodic solution for the following fourth-order *p*-Laplacian generalized neutral differential equation with attractive and repulsive singularities:

$$\bigl(\varphi_{p}\bigl(u(t)-c(t)u\bigl(t-\delta(t)\bigr) \bigr)''\bigr)''+f \bigl(u(t)\bigr)u'(t)+g\bigl(t,u(t)\bigr)=k(t), $$

where *g* has a singularity at the origin. The novelty of the present article is that we show that attractive and repulsive singularities enable the achievement of a new existence criterion of a positive periodic solution through an application of coincidence degree theory. Recent results in the literature are generalized and significantly improved.

## Introduction

In this paper, we consider the existence of a positive periodic solution for the following fourth-order *p*-Laplacian generalized neutral differential equation with singularity:

1.1 $$ { } \bigl(\varphi_{p}\bigl(u(t)-c(t)u\bigl(t-\delta(t) \bigr)\bigr)''\bigr)''+f \bigl(u(t)\bigr)u'(t)+g\bigl(t,u(t)\bigr)=k(t), $$

where $p\geq2$, $\varphi_{p}(u)=|u|^{p-2}u$ for $u\neq0$ and $\varphi_{p}(0)=0$; $f:\mathbb{R}\to\mathbb{R}$ is a continuous function, $|c(t)|\neq1$, for all $t\in[0,T]$, $c, \delta\in C^{2}(\mathbb{R},\mathbb{R})$ and *c*, *δ* are *T*-periodic functions for some $T > 0$, $\delta'(t)<1$ for all $t\in[0,T]$; $k:\mathbb{R}\rightarrow\mathbb{R}$ is continuous periodic functions with $k(t+T)\equiv k(t)$ and $\int^{T}_{0}k(t)\,dt=0$; $g(t,u)=g_{0}(u)+g_{1}(t,u)$, $g_{1}:\mathbb{R}\times(0,+\infty)\to\mathbb {R}$ is an $L^{2}$-Carathéodory function and $g_{1}(t,\cdot)=g_{1}(t+T,\cdot)$; $g_{0}:(0,+\infty)\to \mathbb{R}$ is a continuous function. *g* can come with a singularity at the origin, i.e.,

$$ \lim_{u\rightarrow0^{+}} g(t,u)=+\infty \quad\Bigl(\mbox{or } \lim _{u\rightarrow0^{+}} g(t,u)=-\infty\Bigr),\quad\mbox{uniformly in } t. $$

It is said that (1.1) is of repulsive type (resp. attractive type) if $g\rightarrow+\infty$ (resp. $g\rightarrow-\infty$) as $u\rightarrow0^{+}$.

In recent years, the study of periodic solutions for neutral differential equations has attracted the attention of many researchers; see [2–9, 14, 16–18, 20, 21] and the references cited therein. For related books, we refer the reader to [1, 12]. Most work concentrated on the neutral operator $(A_{1}u)(t):=u(t)-cu(t-\delta)$ (see [6, 7, 14, 16, 21]) or the neutral operator with variable parameter $(A_{2}u)(t):=u(t)-c(t)u(t-\delta)$ (see [3, 8]) or the neutral operator with variable delay $(A_{3}u)(t):=u(t)-cu(t-\delta(t))$ (see [4, 5]). However, the study of a neutral operator with linear autonomous difference operator $(Au)(t):=u(t)-c(t)u(t-\delta(t))$ is relatively rare.

At the same time, some authors began to consider neutral differential equations with repulsive singularity [11, 13, 23]. Kong et al. [11] in 2015 discussed the following second-order neutral differential equation with repulsive singularity:

1.2 $$ { } \bigl(u(t)-cu(t-\delta)\bigr)''+f \bigl(u(t)\bigr)u'(t)+g\bigl(t,u(t-\tau)\bigr)=e(t), $$

where *c* is a constant with $|c|<1$, *g* allowed is to be repulsive singular at $u=0$. By applying Mawhin’s continuation theorem, the authors have shown that (1.2) had at least one positive *T*-periodic solution. The authors employed an interesting technique dealing with the singularity of $g(t,u)$ at $u=0$. Afterwards, Xin and Cheng [23] in 2017 investigated a kind of second-order neutral differential equation with repulsive singularity:

1.3 $$ { } \bigl(u(t)-cu(t-\delta)\bigr)''+f \bigl(t,u'(t)\bigr)+g\bigl(t,u(t)\bigr)=e(t), $$

where $|c|\neq1$ and *g* had a repulsive singular at $u=0$. The authors found that the existence of positive *T*-periodic solution for (1.3) by applications of coincidence degree theory.

All the aforementioned results are related to neutral differential equations or neutral differential equations with repulsive singularity. Naturally, a new question arises: how does the neutral differential equation with linear autonomous difference operator work on attractive and repulsive singularities? Besides practical interests, the topic has obvious intrinsic theoretical significance. To answer this question, in this paper, we try to establish the existence of periodic solutions of (1.1) by employing coincidence degree theory. The techniques used are quite different from that in [11, 13, 23] and our results are more general than those in [11, 13, 23] in two aspects. Firstly, we first analyze qualitative properties of the neutral operator with a linear autonomous difference operator $(Au)(t)$ in the case that $|c|>1$. Secondly, an attractive singularity is in contradiction with the repulsive singularity. Therefore, the above methods of [11, 13, 23] are no longer applicable to a study of (1.1) with an attractive singularity. So we need to find a new method.

The paper is organized as follows: In Sect. 2, we first analyze qualitative properties of the neutral operator $(Au)(t)$ in the case that $|c|>1$, which will be helpful for further studies of differential equations with this neutral operator. In Sect. 3, we get existence results of positive *T*-periodic solution for (1.1) with repulsive singularity. In Sect. 4, we investigate the existence of a positive *T*-periodic solution for (1.1) with attractive singularity. In Sect. 5, we illustrate our results with a numerical example.

## Preliminary lemmas

Firstly, we recall the coincidence degree theory.

### Lemma 2.1

(Gaines and Mawhin [10])

*Suppose that* *X* *and* *Y* *are two Banach spaces*, *and* $L:D(L)\subset X\rightarrow Y$ *is a Fredholm operator with index zero*. *Let* $\Omega\subset X$ *be an open bounded set and* $N:\overline{\Omega}\rightarrow Y $ *be* *L*-*compact on* Ω̅. *Assume that the following conditions hold*:

1. $Lu\neq\lambda Nu$, $\forall u\in\partial\Omega\cap D(L)$, $\lambda\in(0,1)$;
2. $Nu\notin\operatorname{Im} L$, $\forall u\in\partial\Omega \cap\operatorname{Ker} L$;
3. $\deg\{JQN,\Omega\cap\operatorname{Ker} L,0\}\neq0$, *where* $J:\operatorname{Im} Q\rightarrow\operatorname{Ker} L$ *is an isomorphism*.

*Then the equation* $Lu=Nu$ *has a solution in* $\overline{\Omega}\cap D(L)$.

On the other hand, we consider the properties of the neutral operator *A*.

### Lemma 2.2

(see [22])

*If* $|c(t)|<1$, *then the operator* $(Au)(t)$ *has a continuous inverse* $A^{-1}$ *on the space*

$$C_{T}:=\bigl\{ u|u\in(\mathbb{R},\mathbb{R}), u(t+T)\equiv u(t), \forall t\in\mathbb{R}\bigr\} , $$

*satisfying*

$$\bigl\vert \bigl(A^{-1}u \bigr) (t) \bigr\vert \leq \frac{ \Vert u \Vert }{1-c_{\infty}},\quad \textit{for } c_{\infty}:=\max_{t\in[0,T]} \bigl\vert c(t) \bigr\vert < 1\ \forall u\in C_{T}. $$

Next, we study the properties of the neutral operator *A* in the case that $|c(t)|>1$. Firstly, we give the following lemma.

### Lemma 2.3

(see [15])

*If* $c(t)\in C_{T}$, $\delta(t)\in C_{T}^{1}:=\{u\in C^{1}(\mathbb{R},\mathbb{R}): u(t+T)=u(t)\}$ *and* $\delta'(t)<1$, *then* $c(\mu(t))\in C_{\omega}$, *here* $\mu(t)$ *is the inverse function of* $t-\delta(t)$.

### Lemma 2.4

*If* $|c(t)|>1$ *and* $\delta'(t)<1$, *then the operator* *A* *has a continuous inverse* $A^{-1}$ *on* $C_{T}$, *satisfying*

$$ \bigl\vert \bigl(A^{-1}u\bigr) (t) \bigr\vert \leq \frac{ \Vert u \Vert }{c_{0}-1},\quad \textit{for } c_{0}:=\min_{t\in [0,T]} \bigl\vert c(t) \bigr\vert >1\ \forall u\in C_{T}. $$

### Proof

Let $t-\delta(t):=s\in\mathbb{R}$. From Lemma 2.3, there exists a continuous function $\mu:\mathbb{R}\rightarrow\mathbb{R}$ such that $\mu(t-\delta(t))=\mu(s)=t$. Let

$$V:C_{T}\rightarrow C_{T},\quad (Vu) (t):=u\bigl(t-\delta(t) \bigr). $$

Then there exists an operator $V^{-1}:C_{T}\rightarrow C_{T}$ such that $(V^{-1}u)(t-\delta(t))=(V^{-1}u)(s)=u(t)=u(\mu(s))$, i.e., $(V^{-1}u)(t)=u(\mu(t))$.

Let

$$\begin{aligned} &E:C_{T}\rightarrow C_{T},\quad (Eu) (t):=u(t)- \frac{1}{c(t)}u\bigl(\mu(t)\bigr), \\ &B:C_{T}\rightarrow C_{T},\quad (Bu) (t):= \frac{1}{c(t)}u\bigl(\mu(t)\bigr), \end{aligned}$$

and

$$D_{0}=t,\qquad D_{j}=\underbrace{\mu\bigl(\mu\bigl(\mu \bigl(\cdots\mu\bigl(\mu }_{j}(t)\bigr)\bigr)\bigr)\bigr),\quad j=1,2,3,\ldots. $$

By the definition of the linear operator *B*, we can get

$$\begin{aligned} &\bigl(B^{2}u\bigr) (t)=\frac{1}{c(t)}\cdot\frac{1}{c(\mu(t))}u \bigl(\mu\bigl(\mu(t)\bigr)\bigr) =\frac{1}{c(D_{0})}\cdot\frac{1}{c(D_{1})}u(D_{2}), \\ &\cdots \\ &\bigl(B^{j}u\bigr) (t)=\prod^{j}_{i=1} \frac{1}{c(D_{i-1})}u(D_{j}). \end{aligned}$$

Then

$$\sum^{\infty}_{j=0}\bigl(B^{j}u\bigr) (t)=\sum^{\infty}_{j=0}\prod ^{j}_{i=1}\frac {1}{c(D_{i-1})}u(D_{j}) =u(t)+\sum^{\infty}_{j=1}\prod ^{j}_{i=1}\frac{1}{c(D_{i-1})}u(D_{j}). $$

Since $\|B\|<1$, we can see that the operator *E* has a continuous inverse $E^{-1}:C_{\omega}\rightarrow C_{\omega}$, satisfying

$$\bigl(E^{-1}u\bigr) (t)=\bigl((I-B)^{-1}u\bigr) (t)=\sum ^{\infty}_{j=0}\bigl(B^{j}u\bigr) (t). $$

From $(Au)(t)=u(t)-c(t)u(t-\delta(t))$, we have

$$\begin{aligned} (Au) (t)&=u(t)-c(t)u\bigl(t-\delta(t)\bigr) \\ &=-c(t)\biggl[u\bigl(t-\delta(t)\bigr)-\frac{1}{c(t)}u(t)\biggr] \\ &=-c(t)V\biggl[u(t)-\frac{1}{c(t)}u\bigl(\mu(t)\bigr)\biggr] \\ &=-c(t) (VEu) (t). \end{aligned}$$

Then, from the above analysis, we can see that there exists an operator $A^{-1}$, and

$$\begin{aligned} \bigl(A^{-1}u\bigr) (t)&=-\frac{1}{c(t)}\bigl(E^{-1}V^{-1}u \bigr) (t) \\ &=-\frac{1}{c(t)}\bigl(E^{-1}u\bigr) \bigl(\mu(t)\bigr). \end{aligned}$$

Therefore, we have

$$\begin{aligned} \bigl\vert \bigl(A^{-1}u\bigr) (t) \bigr\vert &= \biggl\vert - \frac{1}{c(t)}\bigl(E^{-1}u\bigr) \bigl(\mu(t)\bigr) \biggr\vert = \Biggl\vert \frac{1}{c(t)}\sum^{\infty}_{j=0} \bigl(B^{j}u\bigr) \bigl(\mu(t)\bigr) \Biggr\vert \\ &= \Biggl\vert \frac{1}{c(t)} \Biggl(u\bigl(\mu(t)\bigr)+\sum ^{\infty}_{j=1}\bigl(B^{j}u\bigr) \bigl(\mu(t) \bigr) \Biggr) \Biggr\vert \\ &\leq\frac{1}{c_{0}} \Biggl\vert u\bigl(\mu(t)\bigr)+\sum ^{\infty}_{j=1}\prod^{j}_{i=1} \frac{1}{c(D_{i})}u(D_{j+1}) \Biggr\vert \\ &\leq\frac{1}{c_{0}} \Biggl(1+\sum^{\infty}_{j=1} \biggl(\frac{1}{c_{0}} \biggr)^{j} \Biggr) \Vert u \Vert \\ &\leq\frac{ \Vert u \Vert }{c_{0}-1}. \end{aligned}$$

 □

Next, we rewrite (1.1) in the form

2.1 $$ { } \textstyle\begin{cases} (Au_{1})'(t)=u_{2}(t),\\ u_{2}'(t)=\varphi_{q}(u_{3}(t)),\\ u_{3}'(t)=u_{4}(t),\\ u_{4}'(t)=-f(u_{1}(t))u_{1}'(t)-g(t,u_{1}(t))+k(t), \end{cases} $$

where $\frac{1}{p}+\frac{1}{q}=1$. Clearly, if $u(t)=\operatorname{col}(u_{1}(t),u_{2}(t),u_{3}(t),u_{4}(t))$ is periodic solution for (2.1), then $u_{1}(t)$ must be a periodic solution for (1.1). Therefore, the problem of finding an *T*-periodic solution for (1.1) reduces to finding one for (2.1).

Set $X=Y=\{u=\operatorname{col}(u_{1}(t),u_{2}(t),u_{3}(t),u_{4}(t))\in C^{1}(\mathbb{R},\mathbb{R}^{4}): u(t+T)\equiv u(t)\}$ with the norm $\|u\|=\max\{\|u_{1}\|,\|u_{2}\|,\|u_{3}\|,\|u_{4}\|\}$. Clearly, *X* and *Y* are both Banach spaces. Meanwhile, define

$$L:D(L)=\bigl\{ u\in C^{1}\bigl(\mathbb{R},\mathbb{R}^{4} \bigr): u(t+T) = u(t), t \in\mathbb {R}\bigr\} \subset X\rightarrow Y $$

by

$$ (Lu) (t)= \begin{pmatrix} (A u_{1})'(t)\\u_{2}'(t)\\ u_{3}'(t)\\u_{4}'(t) \end{pmatrix} $$

and $N: X\rightarrow Y$ by

2.2 $$ { } (Nu) (t)=\begin{pmatrix}u_{2}(t)\\ \varphi_{q}(u_{3}(t))\\ u_{4}(t)\\-f(u_{1}(t))u_{1}'(t)-g(t,u_{1}(t))+k(t) \end{pmatrix}. $$

Then (2.1) can be converted to the abstract equation $Lu=Nu$.

From $\forall u\in \operatorname{Ker} L$, $u=\left(\begin{array}{c} u_{1}\\ u_{2}\\ u_{3}\\ u_{4} \end{array}\right)\in \mathrm{Ker}L$, i.e. $\left \{ \scriptsize{ \begin{array}{l} (u_{1}(t)-c(t)u_{1}(t-\delta(t)))'=0\\ u_{2}'(t)=0, \\ u_{3}'(t)=0, \\ u_{4}'(t)=0, \end{array}} \right . $ we have

$$ \textstyle\begin{cases} u_{1}(t)-c(t)u_{1}(t-\delta(t))=a_{1},\\ u_{2}(t)=a_{2},\\ u_{3}(t)=a_{3},\\ u_{4}(t)=a_{4}, \end{cases} $$

where $a_{1}$, $a_{2}$, $a_{3}$, $a_{4}\in\mathbb{R}$ are constant. Let $\phi(t)\neq0$ is a solution of $u(t)-c(t)u(t-\delta(t))=1$, then $\mathrm{Ker}L=u=\left(\begin{array}{c} a_{1}\phi (t),\\ a_{2}\\ a_{3}\\ a_{4} \end{array}\right)$. From the definition of *L*, one can easily see that

$$\operatorname{Ker} L \cong\mathbb {R}^{4},\qquad \operatorname{Im} L= \left \{y\in Y: \int_{0}^{T} \begin{pmatrix} y_{1}(s)\\ y_{2}(s)\\y_{3}(s) \\y_{4}(s) \end{pmatrix} \,ds= \begin{pmatrix} 0\\ 0\\ 0 \\0 \end{pmatrix} \right \}. $$

So *L* is a Fredholm operator with index zero. Let $P:X\rightarrow \operatorname{Ker} L$ and $Q:Y\rightarrow \operatorname{Im} Q\subset\mathbb {R}^{4}$ be defined by

$$\begin{aligned} &Px= \begin{pmatrix} (Au_{1})(0)\\u_{2}(0)\\ u_{3}(0) \\ u_{4}(0) \end{pmatrix} ;\\ &Qy=\frac{1}{T} \int_{0}^{T} \begin{pmatrix} y_{1}(s)\\ y_{2}(s) \\ y_{3}(s) \\ y_{4}(s) \end{pmatrix} \,ds, \end{aligned}$$

then $\operatorname{Im} P=\operatorname{Ker} L$, $\operatorname{Ker} Q=\operatorname{Im} L$. So, *L* is a Fredholm operator with index zero. Let *K* denote the inverse of $L|_{\operatorname{Ker} p \cap D(L)}$, we have

$$\begin{aligned}{} [Ky](t)={}&\operatorname{col}\biggl( \int^{T}_{0}G_{1}(t,s)y_{1}(s) \,ds, \int ^{T}_{0}G_{2}(t,s)y_{2}(s) \,ds, \\ &{} \int^{T}_{0}G_{3}(t,s)y_{3}(s) \,ds, \int ^{T}_{0}G_{4}(t,s)y_{4}(s) \,ds\biggr), \end{aligned}$$

where

2.3 $$ { } G_{i}(t,s)= \textstyle\begin{cases} \frac{s}{T}, & 0\leq s < t\leq T;\\ \frac{s-T}{T}, & 0\leq t\leq s \leq T; \end{cases}\displaystyle \quad i=1,2,3,n. $$

From (2.2) and (2.3), it is easy to see that *QN* and $K(I-Q)N$ are continuous, $QN(\overline{\Omega})$ is bounded and then $K(I-Q)N(\overline{\Omega})$ is compact for any open bounded $\Omega\subset X$, which means *N* is *L*-compact on Ω̄.

## Periodic solutions for (1.1) with repulsive singularity

In this section, we investigate the existence of positive periodic solution for (1.1) with repulsive singularity. Firstly, we embed Eq. (2.1) into the following equation family with a parameter $\lambda\in(0,1]$:

3.1 $$ { } \textstyle\begin{cases} (Au_{1})'(t)=\lambda u_{2}(t),\\ u_{2}'(t)=\lambda\varphi_{q}(u_{3}(t)),\\ u_{3}'(t)=\lambda u_{4}(t),\\ u_{4}'(t)=-\lambda f(u_{1}(t))u_{1}'(t)-\lambda g(t,u_{1}(t))+\lambda k(t). \end{cases} $$

Substituting $u_{4}(t)=\lambda^{1-p} (\varphi_{p}((Au_{1})''(t)) )'$ into the last equation of (3.1), we can get

3.2 $$ { } \bigl(\varphi_{p}(Au_{1})''(t) \bigr)''+\lambda^{p}f\bigl(u_{1}(t) \bigr)u_{1}'(t)+\lambda ^{p}g \bigl(t,u_{1}(t)\bigr)=\lambda^{p}k(t). $$

### Lemma 3.1

*Suppose the following condition is satisfied*:

$(H_{1})$
*There exist two constants* $0< d_{1}< d_{2}$ *such that* $g(t,u)<0$ *for all* $(t,u) \in[0,T]\times(0,d_{1})$, *and* $g(t,u)>0$ *for all* $(t,u)\in [0,T]\times(d_{2},+\infty)$.

*Then there exists a point* $\tau\in[0,T]$ *such that*

3.3 $$ { } d_{1}\leq u_{1}(\tau)\leq d_{2}. $$

### Proof

Integration of both sides of (3.2) from 0 to *T*, we have

3.4 $$ { } \int^{T}_{0}g\bigl(t,u_{1}(t)\bigr)\,dt=0. $$

From (3.4), there exists a point $\tau\in[0,T]$ such that

$$g\bigl(t_{1},u_{1}(\tau)\bigr)=0. $$

From $(H_{1})$, we can see that (3.3) is satisfied. □

### Lemma 3.2

*Assume that* $c_{\infty}<1$ *and* $(H_{1})$ *hold*. *Suppose the following conditions are satisfied*:

$(H_{2})$
*There exist positive constants* *α*, *β* *such that*
  3.5 $$ { } g(t,u)\leq\alpha u^{p-1}+\beta,\quad \textit{for all } (t,u)\in[0,T]\times(0,+\infty). $$
$(H_{3})$
*There exist two positive constants* *a*, *b* *such that*
  $$\bigl\vert f\bigl(u(t)\bigr) \bigr\vert \leq a \vert u \vert ^{p-2}+b,\quad \forall u\in\mathbb{R}. $$
$(H_{4})$
*We have*
  $$0< \frac{T (aT+2\alpha )}{ (1-c_{\infty}- (\frac{T^{2}}{2\pi}c_{2}+2Tc_{1}+2Tc_{1}\delta_{1}+Tc_{\infty}\delta_{2} +c_{\infty}\delta_{1}^{2}+2c_{\infty}\delta_{1} ) )^{p-1}}< 1, $$
  *where* $\delta_{i}=\max_{t\in[0,\omega]}|\delta^{(i)}(t)|$, $c_{i}=\max_{t\in[0,\omega]}|c^{(i)}(t)|$, $i=1,2$.

*Then there exist positive constants* $M_{1}$, $M_{2}$, $M_{3}$, $M_{4}$ *such that*

3.6 $$ { } u_{1}(t)\leq M_{1}, \qquad \Vert u_{2} \Vert \leq M_{2}, \qquad \Vert u_{3} \Vert \leq M_{3}, \qquad \Vert u_{4} \Vert \leq M_{4}. $$

### Proof

Firstly, we will consider $(Au_{1})''(t)$. Since $(Au_{1})(t)=u_{1}(t)-c(t)u_{1}(t-\delta(t))$, we have

3.7 $$ { } \begin{aligned}[b] (Au_{1})'(t)&= \bigl(u_{1}(t)-c(t)u_{1}\bigl(t-\delta(t)\bigr) \bigr)' \\ &=u_{1}'(t)-c'(t)u_{1}\bigl(t- \delta(t)\bigr)-c(t)u_{1}'\bigl(t-\delta(t) \bigr)+c(t)u_{1}'\bigl(t-\delta (t)\bigr) \delta'(t) \end{aligned} $$

and

$$\begin{aligned} (Au_{1})''(t)={}&\bigl(u_{1}'(t)-c'(t)u_{1} \bigl(t-\delta(t)\bigr)-c(t)u_{1}'\bigl(t-\delta (t) \bigr)+c(t)u_{1}'\bigl(t-\delta(t)\bigr) \delta'(t)\bigr)' \\ ={}&u_{1}''(t)-\bigl[c''(t)u_{1} \bigl(t-\delta(t)\bigr)+c'(t)u_{1}'\bigl(t- \delta(t)\bigr) \bigl(1-\delta '(t)\bigr)+c'(t)u_{1}' \bigl(t-\delta(t)\bigr) \\ &{}+c(t)u_{1}''\bigl(t-\delta(t)\bigr) \bigl(1-\delta'(t)\bigr) \\ &{}-c'(t)u_{1}'\bigl(t-\delta(t)\bigr) \delta'(t)-c(t)u_{1}''\bigl(t- \delta(t)\bigr) \bigl(1-\delta '(t)\bigr)\delta'(t) \\ &{}-c(t)u_{1}'\bigl(t-\delta(t)\bigr) \delta''(t)\bigr] \\ ={}&u_{1}''(t)-c(t)u_{1}'' \bigl(t-\delta(t)\bigr)-\bigl[c''(t)u_{1} \bigl(t-\delta (t)\bigr)+\bigl(2c'(t)-2c'(t) \delta'(t) \\ &{}-c(t)\delta''(t)\bigr)u_{1}' \bigl(t-\delta(t)\bigr) \\ &{}+\bigl(c(t) \bigl(\delta'(t)\bigr)^{2}-2c(t) \delta'(t)\bigr)u_{1}'' \bigl(t-\delta(t)\bigr)\bigr]. \end{aligned}$$

Hence, we can get

$$\begin{aligned} \bigl(Au_{1}''\bigr) (t)={}&(Au_{1})''(t)+c''(t)u_{1} \bigl(t-\delta(t)\bigr)+\bigl(2c'(t)-2c'(t)\delta '(t)-c(t)\delta''(t) \bigr)u_{1}'\bigl(t-\delta(t)\bigr) \\ &{}+\bigl(c(t) \bigl(\delta'(t)\bigr)^{2}-2c(t) \delta'(t)\bigr)u_{1}'' \bigl(t-\delta(t)\bigr). \end{aligned}$$

By applying Lemma 2.2 and $c_{\infty}<1$, we have

$$\begin{aligned} \bigl\Vert u_{1}'' \bigr\Vert &=\max _{t\in[0,T]} \bigl\vert A^{-1}Au_{1}''(t) \bigr\vert \\ &\leq\frac{\max_{t\in[0,T]} \vert Au_{1}''(t) \vert }{1-c_{\infty}} \\ &\leq\frac{\varphi_{q}( \Vert u_{3} \Vert )+c_{2} \Vert u_{1} \Vert +(2c_{1}+2c_{1}\delta_{1}+c_{\infty}\delta_{2}) \Vert u_{1}' \Vert +(c_{\infty}\delta_{1}^{2} +2c_{\infty}\delta_{1}) \Vert u_{1}'' \Vert }{1-c_{\infty}}, \end{aligned}$$

where $c_{i}=\max_{t\in[0,T]}|c^{(i)}(t)|$ and $\delta_{i}=\max_{t\in[0,T]}|\delta^{(i)}(t)|$, $i=1,2$. From (3.3) and the Wirtinger inequality (see [19], Lemma 2.4), we have

3.8 $$\begin{aligned} { } u_{1}(t)&\leq d_{2}+ \int^{T}_{0} \bigl\vert u_{1}'(t) \bigr\vert \,dt \\ &\leq d_{2}+T^{\frac{1}{2}} \biggl( \int^{T}_{0} \bigl\vert u_{1}'(t) \bigr\vert ^{2}\,dt \biggr)^{\frac{1}{2}} \\ &\leq d_{2}+T^{\frac{1}{2}}\frac{T}{2\pi} \biggl( \int^{T}_{0} \bigl\vert u_{1}''(t) \bigr\vert ^{2}\,dt \biggr)^{\frac{1}{2}} \\ &\leq d_{2}+\frac{T^{2}}{2\pi} \bigl\Vert u_{1}'' \bigr\Vert . \end{aligned}$$

From $u_{1}(0)=u_{1}(T)$, there exists a point $t_{2}\in[0,T]$ such that $u_{1}'(t_{2})=0$, then we have

3.9 $$ { } \bigl\Vert u_{1}' \bigr\Vert \leq u_{1}'(t_{2})+ \int^{T}_{0} \bigl\vert u_{1}''(t) \bigr\vert \,dt\leq T \bigl\Vert u_{1}'' \bigr\Vert . $$

Therefore, we have

$$\begin{aligned} & \bigl\Vert u_{1}'' \bigr\Vert \\ &\quad \leq \frac{\varphi_{q}( \Vert u_{3} \Vert )+c_{2} (d_{2}+\frac{T^{2}}{2\pi} \Vert u_{1}'' \Vert )+T(2c_{1}+2c_{1}\delta_{1}+c_{\infty}\delta_{2}) \Vert u_{1}'' \Vert +(c_{\infty}\delta_{1}^{2} +2c_{\infty}\delta_{1}) \Vert u_{1}'' \Vert }{1-c_{\infty}} \\ &\quad \leq \frac{\varphi_{q}( \Vert u_{3} \Vert )+ (\frac{T^{2}}{2\pi }c_{2}+2Tc_{1}+2Tc_{1}\delta_{1}+Tc_{\infty}\delta_{2} +c_{\infty}\delta_{1}^{2}+2c_{\infty}\delta_{1} ) \Vert u_{1}'' \Vert +c_{2}d_{2}}{1-c_{\infty}}. \end{aligned}$$

Since $1-c_{\infty}- (\frac{T^{2}}{2\pi}c_{2}+2Tc_{1}+2Tc_{1}\delta_{1}+Tc_{\infty}\delta_{2} +c_{\infty}\delta_{1}^{2}+2c_{\infty}\delta_{1} )>0$, we have

3.10 $$ { } \bigl\Vert u_{1}'' \bigr\Vert \leq\frac{\varphi_{q}( \Vert u_{3} \Vert )+c_{2}d_{2}}{1-c_{\infty}- (\frac{T^{2}}{2\pi}c_{2}+2Tc_{1}+2Tc_{1}\delta_{1}+Tc_{\infty}\delta_{2} +c_{\infty}\delta_{1}^{2}+2c_{\infty}\delta_{1} )}. $$

On the other hand, from $u_{3}(0)=u_{3}(T)$, there exists a point $t_{4}\in[0,T]$ such that $u_{4}(t_{4})=0$, we have

3.11 $$ { } \begin{aligned}[b] \bigl\vert u_{4}(t) \bigr\vert &\leq \biggl(u_{4}(t_{4})+ \int^{T}_{0} \bigl\vert u_{4}'(t) \bigr\vert \,dt \biggr) \\ &=\lambda \int^{T}_{0} \bigl\vert -f\bigl(u_{1}(t) \bigr)u_{1}'(t)-g\bigl(t,u_{1}(t)\bigr)+k(t) \bigr\vert \,dt \\ &\leq \int^{T}_{0} \bigl\vert f\bigl(u_{1}(t) \bigr) \bigr\vert \bigl\vert u_{1}'(t) \bigr\vert \,dt+ \int^{T}_{0} \bigl\vert g\bigl(t,u_{1}(t) \bigr) \bigr\vert \,dt+ \int^{T}_{0} \bigl\vert k(t) \bigr\vert \,dt. \end{aligned} $$

From $(H_{2})$ and (3.4), we have

3.12 $$ { } \begin{aligned}[b] \int^{T}_{0} \bigl\vert g\bigl(t,u_{1}(t) \bigr) \bigr\vert \,dt&= \int_{g(t,u_{1}(t))\geq0}g\bigl(t,u_{1}(t)\bigr)\,dt- \int _{g(t,u_{1}(t))\leq0}g\bigl(t,u_{1}(t)\bigr)\,dt \\ &=2 \int_{g(t,u_{1}(t))\geq0}g^{+}\bigl(t,u_{1}(t)\bigr)\,dt \\ &\leq2 \int_{g(t,u_{1}(t))\geq0}\bigl(\alpha u_{1}^{p-1}(t)+\beta \bigr)\,dt \\ &\leq2\alpha \int^{T}_{0} \bigl\vert u_{1}(t) \bigr\vert ^{p-1}\,dt+2\beta T, \end{aligned} $$

where $g^{+}(t,u_{1}):=\max\{0,g(t,u_{1})\}$. Substituting (3.8), (3.9) and (3.12) into (3.11), and from $(H_{3})$, we have

3.13 $$\begin{aligned} { } \bigl\vert u_{4}(t) \bigr\vert \leq{}&a \int^{T}_{0} \bigl\vert u_{1}(t) \bigr\vert ^{p-2} \bigl\vert u_{1}'(t) \bigr\vert \,dt+b \int^{T}_{0} \bigl\vert u_{1}'(t) \bigr\vert \,dt \\ &{} +2\alpha \int^{T}_{0} \bigl\vert u_{1}(t) \bigr\vert ^{p-1}\,dt+2\beta T+ \Vert k \Vert T \\ \leq{}&aT \Vert u_{1} \Vert ^{p-2} \bigl\Vert u_{1}' \bigr\Vert +bT \bigl\Vert u_{1}' \bigr\Vert +2T\alpha \Vert u_{1} \Vert ^{p-1}+\bigl(2 \beta+ \Vert k \Vert \bigr) T \\ \leq{}&aT^{2} \biggl(d_{2}+\frac{T^{2}}{2\pi} \bigl\Vert u_{1}'' \bigr\Vert \biggr)^{p-2} \bigl\Vert u_{1}'' \bigr\Vert +bT^{2} \bigl\Vert u_{1}'' \bigr\Vert \\ &{}+ 2T\alpha \biggl(d_{2}+\frac{T^{2}}{2\pi} \bigl\Vert u_{1}'' \bigr\Vert \biggr)^{p-1}+ \bigl(2\beta+ \Vert k \Vert \bigr) T \\ \leq{}&aT^{2} \biggl(1+\frac{2\pi d_{2}}{T^{2} \Vert u_{1}'' \Vert } \biggr)^{p-2} \bigl\Vert u_{1}'' \bigr\Vert ^{p-1}+2T\alpha \biggl(1+\frac {2\pi d_{2}}{T^{2} \Vert u_{1}'' \Vert } \biggr)^{p-1} \bigl\Vert u_{1}'' \bigr\Vert ^{p-1} \\ &{}+bT^{2} \bigl\Vert u_{1}'' \bigr\Vert +\bigl(2\beta+ \Vert k \Vert \bigr) T \\ \leq{}&aT^{2} \biggl(1+\frac{2\pi d_{2}(p-2)}{T^{2} \Vert u_{1}'' \Vert } \biggr) \bigl\Vert u_{1}'' \bigr\Vert ^{p-1}+2T \alpha \biggl(1+\frac{2\pi d_{2}(p-1)}{T^{2} \Vert u_{1}'' \Vert } \biggr) \bigl\Vert u_{1}'' \bigr\Vert ^{p-1} \\ &{}+bT^{2} \bigl\Vert u_{1}'' \bigr\Vert _{\infty}\bigl(2\beta+ \Vert k \Vert \bigr) T \\ ={}& \bigl(aT^{2}+2T\alpha \bigr) \bigl\Vert u_{1}'' \bigr\Vert ^{p-1}+ \biggl(2a\pi d_{2}(p-2)+ \frac{4\pi \alpha d_{2}(p-1)}{T} \biggr) \bigl\Vert u_{1}'' \bigr\Vert ^{p-2} \\ &{}+bT^{2} \bigl\Vert u_{1}'' \bigr\Vert +\bigl(2\beta+ \Vert k \Vert \bigr)T \end{aligned}$$

since $(1+u)^{l}\leq1+(1+l)u$ for $u\in[0,\mu]$, *μ* is a constant. Substituting (3.10) into (3.13), we have

3.14 $$ { } \begin{aligned}[b] \bigl\vert u_{4}(t) \bigr\vert \leq{}& \bigl(aT^{2}+2T\alpha \bigr) \\ &{}\times\frac{ (\varphi_{q}( \Vert u_{3} \Vert )+c_{2}d_{2} )^{p-1}}{ (1-c_{\infty}- (\frac{T^{2}}{2\pi}c_{2}+2Tc_{1}+2Tc_{1}\delta _{1}+Tc_{\infty}\delta_{2} +c_{\infty}\delta_{1}^{2}+2c_{\infty}\delta_{1} ) )^{p-1}} \\ &{}+ \biggl(2a\pi d_{2}(p-2)+\frac{4\pi \alpha d_{2}(p-1)}{T} \biggr) \\ &{}\cdot\frac{ (\varphi_{q}( \Vert u_{3} \Vert )+c_{2}d_{2} )^{p-2}}{ (1-c_{\infty}- (\frac{T^{2}}{2\pi}c_{2}+2Tc_{1}+2Tc_{1}\delta _{1}+Tc_{\infty}\delta_{2} +c_{\infty}\delta_{1}^{2}+2c_{\infty}\delta_{1} ) )^{p-2}} \\ &{}+bT^{2}\frac{\varphi_{q}( \Vert u_{3} \Vert )+c_{2}d_{2}}{ 1-c_{\infty}- (\frac{T^{2}}{2\pi}c_{2}+2Tc_{1}+2Tc_{1}\delta_{1}+Tc_{\infty}\delta_{2} +c_{\infty}\delta_{1}^{2}+2c_{\infty}\delta_{1} )} \\ &{}+\bigl(2\beta+ \Vert k \Vert \bigr) T. \end{aligned} $$

Since $\int^{T}_{0}\varphi_{q}(u_{3}(t))\,dt=\int^{T}_{0}u_{2}'(t)\,dt=0$, then there exists a point $t_{3}\in[0,T]$ such that $u_{3}(t_{3})=0$. From the Wirtinger inequality and (3.14), we can easily get

$$\begin{aligned} \Vert u_{3} \Vert \leq{}& \int^{T}_{0} \bigl\vert u_{4}(t) \bigr\vert \,dt \\ \leq{}& T \Vert u_{4} \Vert \\ \leq{}&T\biggl[ \bigl(aT^{2}+2T\alpha \bigr) \\ &{}\cdot\frac{ (\varphi_{q}( \Vert u_{3} \Vert )+c_{2}d_{2} )^{p-1}}{ (1-c_{\infty}- (\frac{T^{2}}{2\pi}c_{2}+2Tc_{1}+2Tc_{1}\delta _{1}+Tc_{\infty}\delta_{2} +c_{\infty}\delta_{1}^{2}+2c_{\infty}\delta_{1} ) )^{p-1}} \\ &{}+ \biggl(2a\pi d_{2}(p-2)+\frac{4\pi \alpha d_{2}(p-1)}{T} \biggr) \\ &{}\cdot\frac{ (\varphi_{q}( \Vert u_{3} \Vert )+c_{2}d_{2} )^{p-2}}{ (1-c_{\infty}- (\frac{T^{2}}{2\pi}c_{2}+2Tc_{1}+2Tc_{1}\delta _{1}+Tc_{\infty}\delta_{2} +c_{\infty}\delta_{1}^{2}+2c_{\infty}\delta_{1} ) )^{p-2}} \\ &{}+bT^{2}\frac{\varphi_{q}( \Vert u_{3} \Vert )+c_{2}d_{2}}{ 1-c_{\infty}- (\frac{T^{2}}{2\pi}c_{2}+2Tc_{1}+2Tc_{1}\delta_{1}+Tc_{\infty}\delta_{2} +c_{\infty}\delta_{1}^{2}+2c_{\infty}\delta_{1} )} \\ &{}+\bigl(2\beta+ \Vert k \Vert \bigr) T\biggr]. \end{aligned}$$

Therefore, we get

$$\begin{aligned} \Vert u_{3} \Vert \leq{}&T\biggl[ \bigl(aT^{2}+2T\alpha \bigr) \\ &{}\cdot\frac{ \Vert u_{3} \Vert +(p-1) \Vert u_{3} \Vert ^{2-q}c_{2}d_{2}+\cdots+(c_{2}d-2)^{p-1}}{ (1-c_{\infty}- (\frac{T^{2}}{2\pi}c_{2}+2Tc_{1}+2Tc_{1}\delta _{1}+Tc_{\infty}\delta_{2} +c_{\infty}\delta_{1}^{2}+2c_{\infty}\delta_{1} ) )^{p-1}} \\ &{}+ \biggl(2a\pi d_{2}(p-2)+\frac{4\pi \alpha d_{2}(p-1)}{T} \biggr) \\ &{}\cdot\frac{ \Vert u_{3} \Vert ^{2-q}+\cdots+(c_{2}d_{2})^{p-2}}{ (1-c_{\infty}- (\frac{T^{2}}{2\pi}c_{2}+2Tc_{1}+2Tc_{1}\delta _{1}+Tc_{\infty}\delta_{2} +c_{\infty}\delta_{1}^{2}+2c_{\infty}\delta_{1} ) )^{p-2}} \\ &{}+bT^{2}\frac{\varphi_{q}( \Vert u_{3} \Vert )+c_{2}d_{2}}{ 1-c_{\infty}- (\frac{T^{2}}{2\pi}c_{2}+2Tc_{1}+2Tc_{1}\delta_{1}+Tc_{\infty}\delta_{2} +c_{\infty}\delta_{1}^{2}+2c_{\infty}\delta_{1} )} \\ &{}+\bigl(2\beta+ \Vert k \Vert _{\infty}\bigr)T\biggr]. \end{aligned}$$

Since $p\geq2$ and $\frac{T (aT^{2}+2T\alpha )}{ (1-c_{\infty}- (\frac{T^{2}}{2\pi}c_{2}+2Tc_{1}+2Tc_{1}\delta_{1}+Tc_{\infty}\delta_{2} +c_{\infty}\delta_{1}^{2}+2c_{\infty}\delta_{1} ) )^{p-1}}<1$, there exists a positive constant $M_{3}$ (independent of *λ*) such that

3.15 $$ { } \Vert u_{3} \Vert \leq M_{3}. $$

Substituting (3.15) into (3.10), we get

$$\begin{aligned} \bigl\Vert u_{1}'' \bigr\Vert &\leq \frac{\varphi_{q}( \Vert u_{3} \Vert )+c_{2}d_{2}}{1-c_{\infty}- (\frac {T^{2}}{2\pi}c_{2}+2Tc_{1}+2Tc_{1}\delta_{1}+Tc_{\infty}\delta_{2} +c_{\infty}\delta_{1}^{2}+2c_{\infty}\delta_{1} )} \\ &\leq\frac{M_{3}^{q-1}+c_{2}d_{2}}{1-c_{\infty}- (\frac{T^{2}}{2\pi }c_{2}+2Tc_{1}+2Tc_{1}\delta_{1}+Tc_{\infty}\delta_{2} +c_{\infty}\delta_{1}^{2}+2c_{\infty}\delta_{1} )}:=M_{2}^{*}. \end{aligned}$$

Thus, we have

3.16 $$ { } u_{1}(t)\leq d_{2}+\frac{T^{2}}{2\pi} \bigl\Vert u_{1}'' \bigr\Vert \leq d_{2}+\frac{T^{2}}{2\pi}M_{2}^{*}:=M_{1}, $$

and from (3.9), we have

3.17 $$ { } \bigl\Vert u_{1}' \bigr\Vert \leq T \bigl\Vert u_{1}'' \bigr\Vert \leq T M_{2}^{*}:=M_{1}^{*}. $$

Therefore, from (3.7), (3.16) and (3.17), we have

3.18 $$ { } \Vert u_{2} \Vert \leq M_{1}^{*}+c_{1}M_{1}+c_{\infty}M_{1}^{*}(1+\delta_{1}):=M_{2}. $$

On the other hand, from (3.11) and (3.12), we can get

3.19 $$ { } \begin{aligned}[b] \Vert u_{4} \Vert & \leq\max \biggl\vert \int^{T}_{0}u_{4}'(t)\,dt \biggr\vert \leq \lambda \int^{T}_{0} \bigl\vert -f\bigl(u_{1}(t) \bigr)u_{1}'(t)-g\bigl(t,u_{1}(t)\bigr)+k(t) \bigr\vert \,dt \\ &\leq \lambda \biggl( \int^{T}_{0} \bigl\vert f\bigl(u_{1}(t) \bigr) \bigr\vert \bigl\vert u'(t) \bigr\vert \,dt+ \int ^{T}_{0} \bigl\vert g\bigl(t,u_{1}(t) \bigr) \bigr\vert \,dt+ \int^{T}_{0} \bigl\vert k(t) \bigr\vert \,dt \biggr) \\ &\leq \lambda \bigl( \Vert f_{M_{1}} \Vert M_{1}^{*}T +2 \alpha TM_{1}^{p-1}+2\beta T +T \Vert k \Vert \bigr):= M_{4}, \end{aligned} $$

where $\|f_{M_{1}}\|=\max_{0< u_{1}\leq M_{1}}|f(u_{1}(t))|$. □

### Lemma 3.3

*Assume that* $c_{0}>1$ *and* $(H_{1})$*–*$(H_{3})$ *hold*. *Suppose the following conditions are satisfied*:

$(H_{5})$
*We have*
  $$0< \frac{T (aT+2\alpha )}{ (c_{0}-1- (\frac{T^{2}}{2\pi}c_{2}+2Tc_{1}+2Tc_{1}\delta_{1}+Tc_{\infty}\delta_{2} +c_{\infty}\delta_{1}^{2}+2c_{\infty}\delta_{1} ) )^{p-1}}< 1. $$
  *Then there exist positive constants* $M_{1}', M_{2}', M_{3}', \ldots, M_{n}'$ *such that*
  3.20 $$ { } u_{1}(t)\leq M_{1}',\qquad \Vert u_{2} \Vert \leq M_{2}',\qquad \Vert u_{3} \Vert \leq M_{3}',\qquad \Vert u_{4} \Vert \leq M_{4}'. $$

### Proof

We follow the same strategy and notation as in the proof of Lemma 3.2. From $c_{0}>1$ and Lemma 2.4, we have

$$\begin{aligned} \bigl\Vert u_{1}'' \bigr\Vert &=\max _{t\in[0,T]} \bigl\vert A^{-1}Au_{1}''(t) \bigr\vert \\ &\leq\frac{\max_{t\in[0,T]} \vert Au_{1}''(t) \vert }{c_{0}-1} \\ &\leq\frac{\varphi_{q}( \Vert u_{3} \Vert )+c_{2} \Vert u_{1} \Vert +(2c_{1}+2c_{1}\delta_{1}+c_{\infty}\delta_{2}) \Vert u_{1}' \Vert +(c_{\infty}\delta_{1}^{2} +2c_{\infty}\delta_{1}) \Vert u_{1}'' \Vert }{c_{0}-1} \\ &\leq\frac{\varphi_{q}( \Vert u_{3} \Vert )+ (\frac{T^{2}}{2\pi }c_{2}+2Tc_{1}+2Tc_{1}\delta_{1}+Tc_{\infty}\delta_{2} +c_{\infty}\delta_{1}^{2}+2c_{\infty}\delta_{1} ) \Vert u_{1}'' \Vert +c_{2}d_{2}}{c_{0}-1}. \end{aligned}$$

Since $c_{0}-1- (\frac{T^{2}}{2\pi}c_{2}+2Tc_{1}+2Tc_{1}\delta_{1}+Tc_{\infty}\delta_{2} +c_{\infty}\delta_{1}^{2}+2c_{\infty}\delta_{1} )>0$, we have

$$ \bigl\Vert u_{1}'' \bigr\Vert \leq \frac{\varphi_{q}( \Vert u_{3} \Vert )+c_{2}d_{2}}{c_{0}-1- (\frac {T^{2}}{4\pi}c_{2}+\sqrt{T}c_{1}+\sqrt{T}c_{1}\delta_{1}+\frac{\sqrt {T}}{2}c_{\infty}\delta_{2} +c_{\infty}\delta_{1}^{2}+2c_{\infty}\delta_{1} )}. $$

Similarly, we can get $\|u_{3}\|\leq M_{3}'$. □

### Lemma 3.4

*Assume that* $c_{\infty}<1$ *and* $(H_{1})$*–*$(H_{4})$ *hold*. *Furthermore*, *suppose the following repulsive condition is satisfied*:

$(H_{6})$
$\lim_{u\to0^{+}}\int^{1}_{u}g_{0}(s)\,ds=-\infty$.

*Then there exists a positive constant* $M^{*}$ *such that*

3.21 $$ { } u(t)>M^{*},\quad \textit{for all } t\in[0,T]. $$

### Proof

From $g(t,u)=g_{0}(u)+g_{1}(t,u)$, (3.2) is rewritten in the form

3.22 $$ { } \bigl(\varphi_{p}(Au_{1})''(t) \bigr)''+\lambda^{p}f\bigl(u_{1}(t) \bigr)u_{1}'(t)+\lambda ^{p}(g_{0} \bigl(u_{1}(t)\bigr)+g_{1}\bigl(t,u_{1}(t)\bigr)= \lambda^{p} k(t). $$

Let $\tau\in[0,T]$ be as in (3.3), for any $\tau\leq t\leq T$. Multiplying both sides of (3.22) by $u_{1}'(t)$ and integrate on $[\tau, t]$, we have

3.23 $$ { } \begin{aligned}[b] \lambda^{p} \int^{u_{1}(t)}_{u_{1}(\tau)}g_{0}(u)\,du={}& \lambda^{p} \int^{t}_{\tau }g_{0}\bigl(u_{1}(s) \bigr)u_{1}'(s)\,ds \\ ={}&- \int^{t}_{\tau}\bigl(\varphi_{p}(Au_{1})''(s) \bigr)''u_{1}'(s)\,ds- \lambda^{p} \int^{t}_{\tau}f\bigl(u_{1}(s)\bigr) \bigl\vert u_{1}'(s) \bigr\vert ^{2}\,ds \\ &{}-\lambda^{p} \int^{t}_{\tau}g_{1}\bigl(s,u_{1}(s) \bigr)u_{1}'(s)\,ds+\lambda^{p} \int^{t}_{\tau }k(s)u_{1}'(s) \,ds. \end{aligned} $$

By (3.2), (3.12), (3.16) and (3.17), we have

3.24 $$ { } \begin{aligned}[b] & \biggl\vert \int^{t}_{\tau}\bigl(\varphi_{p}(Au_{1})''(s) \bigr)''u_{1}'(s)\,ds \biggr\vert \\ &\quad \leq \int ^{t}_{\tau} \bigl\vert \bigl( \varphi_{p}(Au_{1})''(s) \bigr)'' \bigr\vert \bigl\vert u_{1}'(s) \bigr\vert \,ds \\ &\quad \leq \bigl\Vert u_{1}' \bigr\Vert \int^{T}_{0} \bigl\vert \bigl( \varphi_{p}(Au_{1})''(s) \bigr)'' \bigr\vert \,ds \\ &\quad \leq\lambda^{p} M_{1}^{*} \biggl( \int^{T}_{0} \bigl\vert f\bigl(u_{1}(s) \bigr) \bigr\vert \bigl\vert u_{1}'(s) \bigr\vert \,ds+ \int^{T}_{0} \bigl\vert g\bigl(s,u_{1}(s) \bigr) \bigr\vert \,ds+ \int ^{T}_{0} \bigl\vert e(s) \bigr\vert \,ds \biggr) \\ &\quad \leq \lambda^{p} M_{1}^{*} \bigl( \Vert f_{M_{1}} \Vert M_{1}^{*}T +2\alpha TM_{1}^{p-1}+2T \beta +T \Vert k \Vert \bigr). \end{aligned} $$

Moreover, we have

3.25 $$ { } \begin{aligned} & \biggl\vert \int^{t}_{\tau}f\bigl(u_{1}(s)\bigr) \bigl\vert u_{1}'(s) \bigr\vert ^{2}\,ds \biggr\vert \leq \bigl\Vert u_{1}' \bigr\Vert ^{2} \int ^{T}_{0} \bigl\vert f\bigl(u_{1}(s) \bigr) \bigr\vert \,ds\leq \Vert f_{M_{1}} \Vert M_{1}^{*2}T, \\ & \biggl\vert \int^{t}_{\tau}g_{1}\bigl(s,u_{1}(s) \bigr)u_{1}'(s)\,ds \biggr\vert \leq \bigl\Vert u_{1}' \bigr\Vert \int ^{T}_{0} \bigl\vert g_{1}(t,u(t) \bigr\vert \,dt\leq M_{1}^{*}\sqrt{T} \Vert g_{M_{1}} \Vert _{2}, \\ & \biggl\vert \int^{t}_{\tau}k(s)u_{1}'(s) \vert \,dt \biggr\vert \leq M_{1}^{*}T \Vert k \Vert , \end{aligned} $$

where $g_{M_{1}}:=\max_{0\leq u_{1}\leq M_{1}}|g_{1}(t,u_{1})|\in L^{2}(0,T)$ is as in $(H_{3})$. Substituting (3.24) and (3.25) into (3.23), we have

$$\begin{aligned} \biggl\vert \int^{u_{1}(t)}_{u_{1}(\tau)}g_{0}(u)\,du \biggr\vert & \leq M_{1}^{*} \bigl(2 \Vert f_{M_{1}} \Vert M_{1}^{*}T +2\alpha TM_{1}^{p-1}+2T \beta+ \sqrt{T} \Vert g_{M_{1}} \Vert _{2}+2T \Vert k \Vert \bigr) \\ &:=M_{5}^{*}. \end{aligned}$$

From the repulsive condition $(H_{6})$, it is clear that there exists a constant $M^{*}>0$ such that

3.26 $$ { } u_{1}(t)\geq M^{*},\quad \forall t\in[\tau,T]. $$

The case $t\in[0,\tau]$ can be treated similarly. □

### Lemma 3.5

*Assume that* $c_{0}>1$ *and* $(H_{1})$*–*$(H_{3})$, $(H_{5})$, $(H_{6})$ *hold*. *Then there exists a positive constant* $M'$ *such that*

3.27 $$ u(t)>M',\quad \textit{for all } t\in[0,T]. $$

### Proof

We follow the same strategy and notation as in the proof of Lemma 3.4. □

By Lemma 2.1, 3.1, 3.2, 3.4, we get the following main result.

### Theorem 3.6

*Assume that* $c_{\infty}<1$ *and* $(H_{1})$*–*$(H_{4})$, $(H_{6})$ *hold*. *Then* (1.1) *has at least one positive periodic solution*.

### Proof

From Lemma 3.1, 3.2, 3.4, we have

$$\begin{aligned} \Omega_{2}={}&\bigl\{ u=\operatorname{col}(u_{1},u_{2},u_{3},u_{4}): E^{*}\leq u_{1}(t)\leq E_{1}, \Vert u_{2} \Vert \leq E_{2}, \Vert u_{3} \Vert \leq E_{3}, \Vert u_{4} \Vert \leq E_{4} , \\ &{} \forall t\in[0,T]\bigr\} , \end{aligned}$$

where $0< E^{*}<\min(M^{*}, d_{1})$, $E_{2}>\max(M_{1},d_{2}) $, $E_{2}>M_{2}$, $E_{3}>M_{3}$, $E_{4}>M_{4}$.

$\Omega=\{u:u\in\partial\Omega_{2} \cap \operatorname{Ker} L\}$ then $\forall u\in \partial\Omega\cap \operatorname{Ker} L$,

$$ QNu=\frac{1}{T} \int^{T}_{0} \begin{pmatrix}u_{2}(t)\\ \varphi_{q}(u_{3}(t)) \\ u_{4}(t)\\ -f(u_{1}(t))u_{1}'(t)-g(t,u_{1}(t))+k(t) \end{pmatrix} \,dt. $$

If $QNu=0$, then $u_{1}=E_{1}$, $u_{2}=0$, $u_{3}=0$, $u_{4}=0$. But if $u_{1}(t)=E_{1}$, we know

$$\int^{T}_{0}g(t,E_{1})\,dt=0. $$

From assumption $(H_{1})$, we have $u_{1}(t)\leq d_{2}\leq E_{1}$, which yields a contradiction. We also have $QNu\neq0$, i.e., $\forall u\in\partial\Omega\cap \operatorname{Ker} L$, $u\notin \operatorname{Im} L$, so conditions (1) and (2) of Lemma 2.1 are both satisfied. Next, we consider (3) of Lemma 2.1 to be also satisfied. In fact, from $(H_{1})$, we have

$$g(t,E_{1})< 0\quad \mbox{and}\quad g(t,E_{1})>0. $$

So condition (3) of Lemma 2.1 is satisfied. By application of Lemma 2.1, (1.1) has a positive *T*-periodic solution. □

Similarly, by Lemma 2.1, 3.1, 3.3, 3.5, we have the following theorem.

### Theorem 3.7

*Assume that* $c_{0}>1$ *and* $(H_{1})$*–*$(H_{3})$, $(H_{5})$, $(H_{6})$ *hold*. *Then* (1.1) *has at least one positive periodic solution*.

### Remark 3.8

If (1.1) satisfies attractive singularity, i.e., $\lim_{x\to0^{+}}\int^{1}_{u}g(s)\,ds=+\infty$. Obviously, the attractive condition and $(H_{1})$, $(H_{2})$ are in contradiction. Therefore, the above method is no long applicable to the proof of the existence of a periodic solution for (1.1) with attractive singularity. We have to find another way.

## Periodic solutions for (1.1) with attractive singularity

In this section, we investigate the existence of positive periodic solution for (1.1) with attractive singularity.

### Theorem 4.1

*Assume that* $c_{\infty}<1$ *and* $(H_{3})$, $(H_{4})$ *hold*. *Furthermore*, *suppose the following conditions hold*:

$(H_{7})$
*There exist constants* $0< d_{3}< d_{4}$ *such that* $g(t,u)>0$ *for* $(t,u)\in[0,T]\times(0,d_{3})$ *and* $g(t,u)<0$ *for* $(t,u)\in[0,T]\times(d_{4},+\infty)$.
$(H_{8})$
*There exist positive constants* *α* *and* *β* *such that*
  4.1 $$ { } -g(t,u)\leq\alpha u^{p-1}+\beta,\quad \textit{for all } (t,u)\in[0,T]\times(0,+\infty). $$
$(H_{9})$
(*Attractive singularity*) $\lim_{u\to 0^{+}}\int^{1}_{u}g_{0}(s)\,ds=+\infty$.

*Then* (1.1) *has at least one positive periodic solution*.

### Proof

We follow the same strategy and notation as in the proof of Theorem 3.6. From Lemma 3.1, we know that there exists a point $\xi\in(0,T)$ such that

$$d_{3}\leq u_{1}(\xi)\leq d_{4}. $$

From (3.12) and $(H_{8})$, we have

4.2 $$ { } \begin{aligned}[b] \int^{T}_{0} \bigl\vert g\bigl(t,u_{1}(t) \bigr) \bigr\vert \,dt&= \int_{g(t,u_{1}(t))\geq0}g\bigl(t,u_{1}(t)\bigr)\,dt- \int _{g(t,u_{1}(t))\leq0}g\bigl(t,u_{1}(t)\bigr)\,dt \\ &=-2 \int_{g(t,u_{1}(t))\leq0}g^{-}\bigl(t,u_{1}(t)\bigr)\,dt \\ &\leq2 \int_{g(t,u_{1}(t))\leq0}\bigl(\alpha u_{1}^{p-1}(t)+\beta \bigr)\,dt \\ &\leq2\alpha \int^{T}_{0} \bigl\vert u_{1}(t) \bigr\vert ^{p-1}\,dt+2\beta T, \end{aligned} $$

where $g^{-}(t,u_{1}):=\min\{g(t,u_{1}),0\}$. The remaining part of the proof is the same as Theorem 3.6. □

### Theorem 4.2

*Assume that* $c_{0}>1$ *and* $(H_{3})$, $(H_{5})$, $(H_{7})$*–*$(H_{9})$ *hold*. *Then* (1.1) *has at least one positive periodic solution*.

## Example

We illustrate our results with one numerical example.

### Example 5.1

Consider the following fourth-order neutral nonlinear differential equation with repulsive singularity:

5.1 $$ { } \begin{aligned}[b] & \biggl(\varphi_{p} \biggl(u(t)-\frac{1}{64}\sin(4t)u \biggl(t-\frac{1}{32}\cos 4t \biggr) \biggr)'' \biggr)''+ \frac{1}{25\pi^{2}}u(t)u'(t) \\ &\quad {}+\frac{1}{100\pi }(\cos4t+1)u^{2}(t)-\frac{1}{u^{\kappa}(t)}=4 \sin4t, \end{aligned} $$

where $\kappa\geq1$ and $p=2$. It is clear that $T=\frac{\pi}{2}$, $c(t)=\frac{1}{64}\sin4t$, $\delta(t)=\frac{1}{32}\cos 4t$, $\tau(t)=\cos4t$, $k(t)=\sin4t$, $c_{1}=\max_{t\in[0,T]}|\frac{1}{16}\cos4t|=\frac{1}{16}$, $c_{2}=\max_{t\in[0,T]}|{-}\frac{1}{4}\sin4t|=\frac{1}{4}$, $\delta_{1}=\max_{t\in[0,T]}|{-}\frac{1}{8}\sin 4t|=\frac{1}{8}$, $\delta_{2}=\max_{t\in[0,T]}|{-}\frac{1}{2}\cos 4t|=\frac{1}{2}$. $g(t,u(t))=\frac{1}{50\pi}(\cos4t+1)u^{2}(t)-\frac{1}{u^{\kappa}(t)}$, $\alpha=\frac{2}{50\pi}$, $\beta=1$; $f(u(t))=\frac{1}{25\pi^{2}}u(t)$, here $a=\frac{1}{25\pi^{2}}$, $b=0$. It is obvious that $(H_{1})$–$(H_{3})$, $(H_{6})$ hold. Now we consider the assumption condition $(H_{4})$,

$$\begin{aligned} &\frac{T (aT+2\alpha )}{ (1-c_{\infty}- (\frac{T^{2}}{2\pi}c_{2}+2Tc_{1}+2Tc_{1}\delta_{1}+Tc_{\infty}\delta_{2} +c_{\infty}\delta_{1}^{2}+2c_{\infty}\delta_{1} ) )^{p-1}} \\ &\quad =\frac{\pi (\frac{1}{50\pi}+\frac{4}{50\pi} )}{2 (1-\frac {1}{64}- (\frac{\pi}{8}+\frac{\pi}{16} +\frac{\pi}{64}+\frac{\pi}{256}+\frac{1}{64}\times\frac{1}{64}+\frac {1}{32}\times\frac{1}{8} ) )^{3}} \\ &\quad \approx0.4703< 1. \end{aligned}$$

So, by Theorem 3.6, (5.1) has at least one $\frac{\pi}{2}$-periodic solution.

## Conclusions

In this article we introduce the existence of periodic solution for a fourth-order generalized neutral differential equation with attractive and repulsive singularities. The techniques used are quite different from that in [11, 13, 23] and our results are more general than those in [11, 13, 23] in two aspects. Firstly, we first analyze qualitative properties of the neutral operator with linear autonomous difference operator $(Au)(t)$ in the case that $|c|>1$. Secondly, an attractive singularity is in contradiction with the repulsive singularity. Therefore, the methods of [11, 13, 23] are no long applicable to a study of (1.1) with attractive singularity. So we need to find a new method. In this paper, we discuss the existence of a periodic solution for Eq. (1.1) with attractive and repulsive singularities by applications of the coincidence degree theory. Moreover, in view of the mathematical points, the results satisfying conditions attractive and repulsive singularities are valuable to understand the periodic solution for fourth-order general neutral singular differential equations.
